# Curcumin Lowers the Accelerated Speed of Epileptogenesis by Traumatic Brain Injury

**DOI:** 10.61186/ibj.3978

**Published:** 2023-12-20

**Authors:** Hanieh Jahi, Mansoureh Eslami, Mohammad Sayyah, Fariba Karimzadeh, Melika Alesheikh

**Affiliations:** 1 Department of Physiology, Shahid Beheshti University of Medical Sciences, Tehran, Iran;; 2 Department of Basic Sciences, School of Allied Medical Sciences, Shahid Beheshti University of Medical Sciences, Tehran, Iran;; 3 Department of Physiology and Pharmacology, Pasteur Institute of Iran, Tehran, Iran;; 4 Cellular and Molecular Research Center, Iran University of Medical Sciences, Tehran, Iran;; 5 Department of Pharmacy, Tehran University of Medical Sciences, Tehran, Iran

**Keywords:** Glial fibrillary acidic protein, IL-1β, Post-traumatic epilepsy, Traumatic brain injury

## Abstract

**Background:**

Traumatic brain injury or TBI can underlie epilepsy. Prevention of PTE has been of great interest to scientists. Given the antiepileptic, antioxidant and anti-inflammatory activities of curcumin, we examined whether this compound can affect epileptogenesis in rats after TBI.

**Methods:**

Curcumin was injected once a day for two weeks. TBI was induced in the temporal cortex of anesthetized rats using a CCI device. One day after TBI, PTZ, 35 mg/kg, was injected i.p. every other day until manifestation of generalized seizures. The number of PTZ injections was then recorded. Moreover, the extent of cortical and hippocampal IL-1β and GFAP expression in the epileptic rats were measured by Western blot analysis.

**Results:**

Curcumin 50 and 150 mg/kg prevented the development of kindling, whereas TBI accelerated the rate of kindling. Curcumin 20 mg/kg prohibited kindling facilitation by TBI, and reduced the expression of IL-1β and GFAP induced by TBI.

**Conclusion:**

Curcumin can stop the acceleration of epileptogenesis after TBI in rats. Inhibiting hippocampal and cortical overexpression of IL-1β and GFAP seems to be involved in this activity.

## INTRODUCTION

Epileptogenesis is a process of progressive hyperexcitability, which is initiated by an initial insult to the brain, leading to epilepsy^[^^1^^,^^2^^]^. TBI underlies cellular damage and subsequent symptomatic epilepsy^[^^3^^-^^5^^] ^and PTE^[^^6^^]^. The innate immune system reacts to TBI, primarily by producing inflammatory cytokines^[^^1^^]^. Those epilepsies, which are established after TBI, are hardly controlled by antiepileptic drugs^[^^6^^]^. Yet it is not exactly known how neural injury leads to epilepsy^[^^5^^,^^7^^]^. 

Neuroinflammation is one of the well-known and important sequelae after TBI, which triggers the development of epilepsy^[^^6^^]^. Molecular and cellular mechanisms of PTE have widely been investigated; however, finding efficient treatments for PTE continues to be a challenge^[^^8^^]^.

Curcumin is the main ingredient of turmeric with a wide range of pharmacologic activities such as antiseizure, anti-inflammatory, antioxidant, anti-amyloid, anticancer, and neuroprotective effects^[^^1^^,^^9^^,^^10^^]^. The antiepileptic effect of curcumin and the underlying mechanisms are not completely disclosed^[^^1^^,^^11^^,^^12^^]^. Therefore, we aimed to investigate the effect of curcumin pretreatment on epileptogenesis after TBI. We also assessed the effect of this compound on the expression of IL-1β and GFAP in the hippocampal and cortical regions of rats with TBI.

## MATERIALS AND METHODS

**Experimental animals**

 Adult male Wistar rats (250–280 g; n = 120) from Pasteur Institute of Iran (Tehran) were housed under standard laboratory conditions (12:12 h light/dark cycle; 24 ± 1°C) with free access to standard rodents’ food and water ad libitum. 

**Curcumin pretreatment**

Curcumin (Sigma-Aldrich, USA) was dissolved in DMSO (Sigma-Aldrich) and injected once a day for 14 consecutive days via i.p. route at doses of 20, 50, and 150 mg/kg/day. These doses were selected based on a previous study indicating antiepileptic effect of curcumin in mice^[^^11^^]^. 

**TBI induction**

Rats were anesthetized with i.p. injection of chloral hydrate (350 mg/kg; Sigma Aldrich, Canada) and fixed in a stereotaxic device. A circular piece of skull with 5 mm diameter was removed from the left parieto-temporal cortex (coordinates; A, −4 mm from bregma; L, −4 mm from bregma) with no harm to dura. Rats were concussed using a CCI device (AmScien Instruments, Model AMS 201, AmSci, USA) with 5 mm round tip, 4.5 m/s velocity, and 150 ms duration. After the induction of TBI, the dissected skull was returned to its position and fixed with dental acrylic, and the skin was closed. The animals were then returned to clean cages to recover from the surgery. The sham-operated rats underwent only craniotomy without TBI. 

**Chemical kindling**

PTZ (Sigma-Aldrich, Canada) was injected i.p. in a sub-convulsive dose of 35 mg/kg every other day until rats showed one of the scores 4 or 5 seizures in three consecutive trials. Seizure scores were defined according to a previous classification^[^^13^^]^. Clonic convulsions in the forelimbs along with rearing was considered score 4. Generalized colonic convulsions accompanied by falling was considered score 5.

**Histology**

***TTC staining***

The extent of brain injury was measured by TTC (Sigma-Aldrich, Canada) staining according to a previously described method^[^^14^^]^. Brain of each of the anesthetized rats was dissected out. A 2-mm coronal sections were made using a rat matrix and shaving razor. The slices were placed promptly in a 2% (w/v) TTC at 37 °C temperature for 10 min. The area with no TTC staining was considered as damaged area. The depth of injury was measured by a micrometer.

***Immunoblotting assay***

After behavioral experiments, anesthetized rats were perfused using 4% paraformaldehyde in PBS. The cortical and hippocampal areas were dissected manually. Tissues were lysed by cell lysis buffer containing a protease inhibitor cocktail. After centrifuging at 10000 ×g for 15 min, protein concentration of the supernatant was determined by Bradford assay according to company guideline (Bio-Rad, UK). After boiling in Laemmli sample buffer at 95°C for 5 min, the total protein was separated using SDS-PAGE 8% and transferred onto PVDF (GE Healthcare, UK) membrane. Then the membrane was blocked using 5% milk powder in TBST and incubated with rabbit monoclonal antibodies against GFAP and/or IL-1β at 4° C overnight. After rinsing three times with PBS, the membrane was incubated with horseradish peroxidase-conjugated anti-rabbit secondary antibodies at room temperature for 3 h. Finally, the bands were detected by ECL detection kit (GE Healthcare) and quantified by ImageJ software (Broken Symmetry Software Company, USA).

**Experimental groups**

Rats were assigned to 15 groups with 8 animals in each. Detail of the experimental design is described in Table 1. 

**Statistical analysis**

Statistical analyses were performed using Graph Pad Prism 9 (GraphPad Software, CA, USA). Shapiro–Wilk test was used to assess the normal distribution of data. Significant difference among groups was detected using one-way analysis of variance (ANOVA) and Tukey-Kramer multiple comparisons test. Results were expressed as mean ± SEM, and *p* values less than 0.05 were considered statistically significant.

**Table 1:** Experimental design of the study

** Experimental group** ** (n = 8 in each group)**	**Experiment**
DMSO + PTZ kindling	Effect of curcumin on kindling
Curcumin 20 mg/kg + PTZ kindling	
Curcumin 50 mg/kg + PTZ kindling	
Curcumin 150 mg/kg + PTZ kindling	
PTZ kindling	Effect of TBI on kindling
Surgery + PTZ kindling	
TBI + PTZ kindling	
DMSO + TBI + PTZ kindling	Effect of curcumin on kindling in TBI state
Curcumin 20 mg/kg + TBI + PTZ kindling	
DMSO	Histology
Curcumin 20 mg/kg	
DMSO + surgery	
Curcumin 20 mg/kg + surgery	
DMSO + TBI	
Curcumin 20 mg/kg + TBI	

## RESULTS

**Mild cortical injury**

**Fig. 1:**
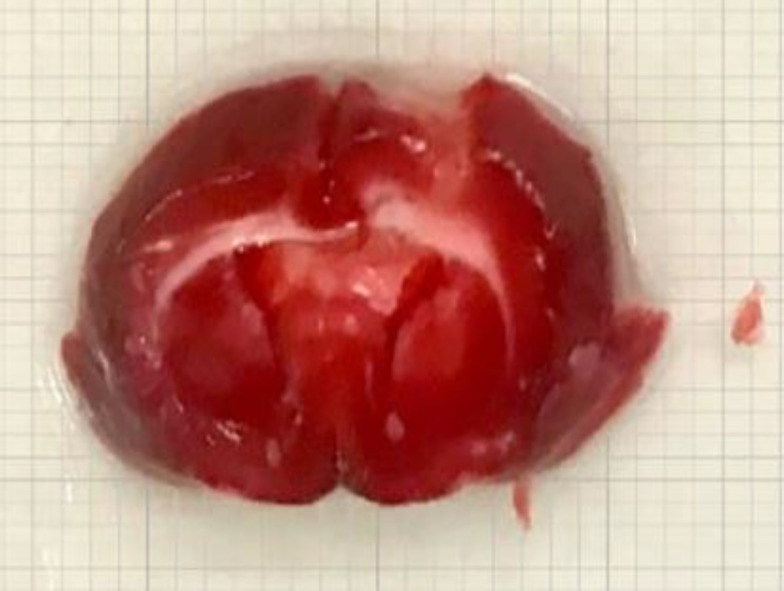
The representative image of cortical tissue damage induced by CCI apparatus

TBI caused a cortical injury in rats with 2 ± 0.5 mm depth from dura (Fig. 1). This finding confirmed the presence of a mild brain injury.

**Effect of controlled cortical injury on PTZ kindling**

**Fig. 2:**
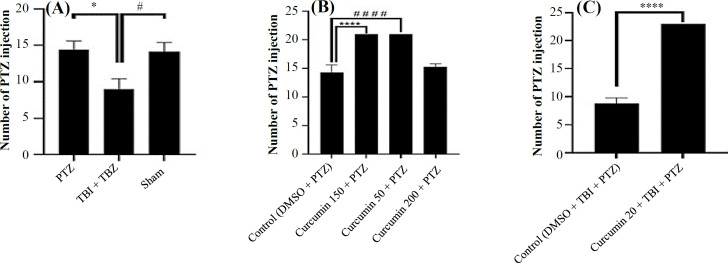
Effect of curcumin on the acquisition of the kindled seizures in rats with TBI. Effect of (A) TBI, (B) curcumin, and (C) curcumin along with TBI on the mean number of PTZ injections required to achieve generalized kindled seizures. Data are presented as mean ± SEM. ^*^p < 0.05; ^****^p < 0.0001; ^####^*p* < 0.0001

Rats with TBI needed less number of PTZ injections to be kindled (*p* < 0.05). The sham-operated and naïve control (PTZ) groups showed a similar number of PTZ injections to reach the kindled state (Fig. 2A).

**Effect of curcumin pretreatment on PTZ kindling**

Curcumin 50 and 150, but not 20 mg/kg/day, increased the number of PTZ injections required for acquisition of generalized seizures (*p* < 0.0001 compared to control group; Fig. 2B). 

**Effect of curcumin on kindling after TBI**

The injured rats pretreated with curcumin 20 mg/kg showed a similar number of PTZ injections to the control non-injured rats. The difference between the curcumin-pretreated and -untreated rats suffering from TBI was extremely significant (*p* < 0.0001 compared to control group; Fig. 2C).

**Effect of curcumin on GFAP expression**

**Fig. 3:**
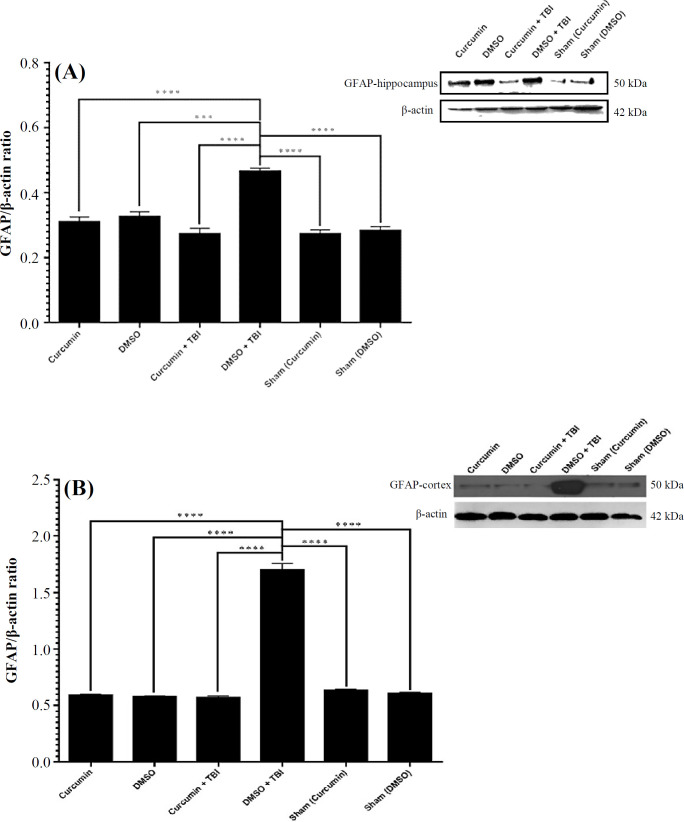
Effect of curcumin 20 mg/kg on GFAP expression in the (A) hippocampus and (B) cortex of rats underwent TBI. DMSO was used as the solvent of curcumin. The results are expressed as mean ± SEM. ^***^p < 0.001; ^****^p < 0.0001

Western blot analysis showed that GFAP expression signiﬁcantly increased in the hippocampus (*p* < 0.0001; Fig. 3A) and cortex (*p* < 0.0001; Fig. 3B) of rats with TBI (DMSO + TBI) compared to the sham, curcumin, and DMSO groups. Expression of GFAP was signiﬁcantly lower in the hippocampus (*p* < 0. 0.0001; Fig. 3A) and cortex (*p* < 0. 0.0001; Fig. 3B) of trauma-injured rats pretreated with curcumin, compared to the DMSO + TBI group. 

**Effect of curcumin on IL-1β** **expression**

**Fig. 4:**
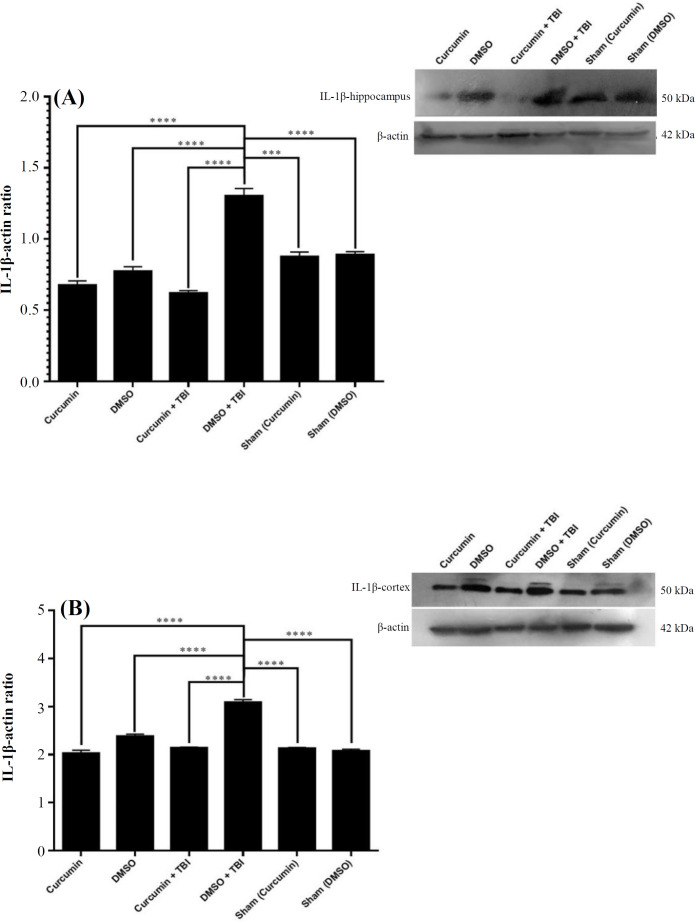
Effect of curcumin 20 mg/kg on IL-1β expression in the (A) hippocampus and (B) cortex (B) of rats with TBI. DMSO was used as the solvent of curcumin. The results are expressed as mean ± SEM. ^****^p < 0.0001

The expression of IL-1β signiﬁcantly increased in the hippocampus (*p* < 0.0001; Fig. 4A) and cortex (*p* < 0.001; Fig. 4B) of the trauma-injured rats (DMSO + TBI) compared to the sham, curcumin, and DMSO groups. However, its expression signiﬁcantly decreased in the hippocampus (*p *< 0.0001; Fig. 4A) and cortex (*p* < 0.0001; Fig. 4B) of curcumin-pretreated injured rats compared to DMSO + TBI group. 

## DISCUSSION

Results of the present study indicated that TBI in the cortex of rats increased the speed of kindling development. Injecting curcumin 20 mg/kg once a day for 14 consecutive days did not change the rate of kindling in sham-operated rats. However, it suppressed the increased rate of kindling in rats with TBI. 

CCI is a famous animal model of TBI with pathological changes similar to clinical cases of TBI^[^^15^^]^. Therefore, it has acceptable applicability in PTE studies^[^^16^^,^^17^^]^. Neuroinflammation is an essential factor in development of seizure activity after brain insults^[^^6^^]^. Drugs or strategies that could interfere with neuroinflammation can prevent or reduce development of seizures^[^^6^^]^.

In our study, chronic administration of curcumin 50 and 150 mg/kg (but not 20 mg/kg) inhibited PTZ kindling. Moreover, curcumin 20 mg/kg inhibited the acceleration of PTZ kindling in rats with TBI. Protective effects of curcumin on TBI, stroke, epilepsy, memory deficit, oxidative stress, neuronal damage, and acute excitotoxicity have previously been reported in various studies^[^^18^^,^^19^^]^. Curcumin is a nontoxic compound that easily passes through BBB^[^^1^^]^. This compound has shown antiseizure activity in chemical and electrical seizure models in animals^[^^10^^]^. In addition to antioxidant and anti-inflammatory effects^[^^20^^-^^22^^]^, curcumin has demonstrated antiepileptic activities in kainate model of temporal lobe epilepsy^[^^12^^]^ and PTZ kindling model^[^^11^^]^. These properties might underlie the antiepileptic effect of curcumin, which is observed in our study in TBI state. 

We found in the present study that TBI significantly increased the expression of GFAP and IL-1β in the hippocampus and cortex of rats. Following TBI, the inflammatory reactions, microglia and astrocytes activation, and release of neuroinflammatory factors initiate^[^^23^^,^^24^^]^. TBI stimulates the secretion of the inflammatory cytokines, mainly IL-1β, which is a critical inflammatory mediator involved in PTE^[^^24^^]^. IL-1β is also involved in microglia/astrocyte function, distraction of BBB, and cytokine release^[^^25^^]^. Within a period after TBI, astrocytes are activated, and a high expression of GFAP is observed^[^^24^^]^. Meanwhile, overactivity of immune cells and cytokines have been observed in the brain foci of seizures^[^^25^^]^. 

Neuroinflammation is involved in seizure initiation and propagation. Therefore, we investigated the effect of curcumin pretreatment on the expression levels of GFAP and IL-1β in the hippocampus and cortex of rats with TBI. Curcumin significantly decreased the overexpression of hippocampal and cortical GFAP and IL-1β in the rats suffered from TBI. The positive role of IL-1β and GFAP in epileptogenesis is well described^[^^26^^-^^30^^]^. Therefore, downregulation of IL-1β and GFAP seems to play a role in the inhibitory effect of curcumin on kindling speed after TBI. 

## CONCLUSION

Curcumin can stop the acceleration of epileptogenesis after TBI in rats. Preventing hippocampal and cortical overexpression of IL-1β and GFAP seems to be among the mechanisms involved in this activity. In order to further disclose potential efficiency of curcumin against TBI complications including epilepsy, the activity of this compound needs to be assessed in animal models of PTE. 

## DECLARATIONS

### Acknowledgments

Authors declare that no artificial intelligence (AI)-assisted technologies has been used in the production of the submitted work.

### Ethical approval

All the experimental procedures in this study were conducted in accordance with the Research Ethics Committee of the European Communities Council Directive of 24 November 1986 (86/609/EEC) and approved by the Institutional Animal Care guidelines of Ethics Committee of Shahid Beheshti University of Medical Sciences, Tehran, Iran (ethical code: IR.SBMU.RETECH.REC.1397.478.).

### Consent to participate

Not applicable.

### Consent for publication

All authors reviewed the results and approved the final version of the manuscript.

### Authors’ contributions

HJ: performing laboratory assessments, collecting samples, and analyzing the data; ME: analyzing the data, writing, reviewing and editing the original draft, and obtaining the funding; MS: supervising the study, conducting experimental design, and reviewing/editing the revised version of the manuscript; FK: performing laboratory assessments and analyzing the data; MA: writing, reviewing and editing the original draft.

### Data availability

All relevant data can be found within the manuscript. 

### Competing interests

The authors declare that they have no competing interests. 

### Funding

This study was supported by School of Allied Medical Sciences, Shahid Beheshti University of Medical Sciences, Tehran, Iran [grant number12680].

### Supplementary information

The online version does not contain supplementary material. 
